# A Review of the Use of Carbon Nanotubes and Graphene-Based Sensors for the Detection of Aflatoxin M1 Compounds in Milk

**DOI:** 10.3390/s21113602

**Published:** 2021-05-21

**Authors:** Jingrong Gao, Shan He, Anindya Nag, Jonathan Woon Chung Wong

**Affiliations:** 1 School of Food Science and Engineering, South China University of Technology, Guangzhou 510640, China; gaojingrong@scut.edu.cn; 2 Institute for NanoScale Science and Technology, College of Science and Engineering, Flinders University, Bedford Park 5042, Australia; 3 School of Chemistry and Chemical Engineering, Guangzhou University, Guangzhou 510006, China; 4 School of Information Science and Engineering, Shandong University, Jinan 251600, China; 5 Institute of Bioresource and Agriculture, Hong Kong Baptist University, 224 Waterloo Road, Kowloon Tong 999077, Hong Kong, China; jwcwong@hkbu.edu.hk

**Keywords:** carbon nanotubes, graphene, immunoassay, aflatoxin M1, aflatoxin B1

## Abstract

This paper presents a comprehensive review of the detection of aflatoxin compounds using carbon allotrope-based sensors. Although aflatoxin M1 and its derivative aflatoxin B1 compounds have been primarily found in milk and other food products, their presence above a threshold concentration causes disastrous health-related anomalies in human beings, such as growth impairment, underweight and even carcinogenic and immunosuppressive effects. Among the many sensors developed to detect the presence of these compounds, the employment of certain carbon allotropes, such as carbon nanotubes (CNTs) and graphene, has been highly preferred due to their enhanced electromechanical properties. These conductive nanomaterials have shown excellent quantitative performance in terms of sensitivity and selectivity for the chosen aflatoxin compounds. This paper elucidates some of the significant examples of the CNTs and graphene-based sensors measuring Aflatoxin M1 (ATM1) and Aflatoxin B1 (AFB1) compounds at low concentrations. The fabrication technique and performance of each of the sensors are shown here, as well as some of the challenges existing with the current sensors.

## 1. Introduction

The utilization of carbon allotropes in the sensing world has brought about revolutionary changes in recent times. After the popularization of sensors at the end of the 20th century, various MEMS [1,2] and printed [3,4] sensors have been fabricated and utilized in different applications. The semiconducting sensors hold the highest priority due to their high accuracy, high linearity, high signal-to-noise ratio and easy interfacing with the control systems [5,6]. The materials used to form these semiconducting sensors have evolved. Earlier, during the 1990s, silicon became the most popular material for forming the substrates of the sensors. This was due to its high stability and repeatability in the responses, the small size of the prototypes and ability to work in extreme conditions [7]. While silicon became a standard material to form the substrates until printed sensors came into the picture, the conductive elements used to form the electrodes were constantly changing. This is due to the direct relationship between the properties of the electrodes with that of the quality of the sensors. With the constant growth in nanotechnology [8,9,10], nanomaterials of various forms, including nanoparticles, nanobeads, nanosheets, nanopowders and quantum dots have been exploited to fabricate the electrodes. Although all of these materials are significant in enhancing the efficiency of the resultant devices, the electrical, mechanical and thermal attributes associated with each of them can differ based on their size, shape and dimensions.

Nanomaterials can be broadly classified into two categories, namely metallic nanoparticles [11,12] and carbon-based allotropes. Among the former group, certain elements such as gold [13,14], silver [15,16], copper [17,18] and aluminum [19,20] have been extensively used in forming the electrodes. The deployment of these metallic elements has been mostly done using the conventional microelectrochemical systems (MEMS) technique [21]. Although all these nanomaterials have been utilized to develop sensors for a wide range of applications, biocompatibility is one critical parameter that needs approval before the use of the sensors for the healthcare sector. The biocompatibility of the sensor may decide the standard of the sensor and affect the condition of the patient. For example, suppose soluble silver compounds are used to form flexible sensors that are used as catheters. In that case, it can lead to certain toxic effects such as liver and kidney damage, irritation of the eyes and respiratory problems [22]. This biocompatibility can be addressed by using specific elements such as carbon-based allotropes. This category includes elements such as carbon nanotubes (CNTs) [23,24,25], graphene [26,27,28], graphite [29,30,31] and fullerenes [32,33,34]. Among these elements, CNTs and graphene have been extensively used for a wide range of applications. The biocompatibility of these elements depends strongly on certain factors such as mass, purity, ratio and surface functional groups [35]. In cases of electrochemical applications as the one shown in the paper, the functionalization of CNTs, graphene and their derivatives has increased the attention towards other nanomaterials to increase the biocompatibility towards biosensing applications. The surface modification also increases the dispersibility of these carbon-based structures as a result of the alteration of cellular interaction pathways. This decreases the cytotoxic effects of CNTs and graphene are tested in vitro conditions before the deployment in real-time scenarios [36].

Each of the above-mentioned conductive elements has been deposited and embedded either on silicon or a range of polymers such as polydimethylsiloxane (PDMS) [37,38], polyethylene terephthalate (PET) [39,40], poly(3,4-ethylene dioxythiophene) polystyrene sulfonate (PEDOT: PSS) [41,42] and polyimide (PI) [43,44]. The sensors formed with these elements have been used in a wide range of environmental [45,46,47] and industrial [48,49,50] sectors. Initially, CNTs were popularized in the late 90s for developing sensors for academic and industrial applications [36,51,52]. CNTs were synthesized in various forms based on the number of walls present in their structures. Two of the common forms, including single-walled carbon nanotubes (SWCNTs) [53,54] and multi-walled carbon nanotubes (MWCNTs) [55,56], have been considered for application purposes. Apart from these two, there are double-walled carbon nanotubes (DWCNTs) [57,58] and few-walled carbon nanotubes (FWCNTs) [59,60], but these are mostly used for characterization purposes. Some of the synthesis processes used to develop CNTs are chemical vapor deposition (CVD) [61,62], arc-discharge [63,64], laser ablation [65,66] and liquid electrolysis [67,68]. The selection of each of these processes depends on the type of CNTs required for the chosen application. Graphene has been proclaimed to be a magic material since its synthesis on a research scale [69]. Scientists have tried fabricating graphene in various ways to increase its availability at a reduced cost. Some of the common techniques of synthesizing graphene are CVD [70,71], Hummers’ method [72,73] and laser ablation of commercial films [74,75]. Graphene has been utilized for a wider sector of applications in comparison to CNTs due to its enhanced electromechanical properties. It has been used in pure and composite [76,77] forms to build robust and highly sensitive sensors. Table 1 [78] shows a comparison between CNTs and graphene in terms of their physicochemical properties. It is seen that both these elements have been highly efficient in the sensing world. This paper showcases the use of sensors based on these elements to detect a particular chemical found in food products.

The use of sensors for electrochemical sensing has been a cornerstone in recent times. Different kinds of MEMS and printed prototypes have been used to detect chemical compounds present in solutions at varied concentrations. The use of CNTs and graphene in sensors for electrochemical sensing has been very efficient due to their high electrical conductivity and charge carrier densities. Out of the applications related to electrochemical sensing, the detection of ions present in food is critical, as a slight aberration in the concentration of the chemical from its optimized value can increase the toxicity of the food product. Among them, aflatoxin M1 (ATM1) is one of the chemical compounds from a group and species of mycotoxins and aspergillus, respectively, that is found in milk and other food products [79,80,81]. While this chemical is primarily present in animal milk, the increase in its concentration can lead to adverse effects on the central nervous system, liver, kidney. It may even cause death [82]. Thus, despite the pasteurization and sterilization of the milk before its consumption, it is important to determine the precise concentration of ATM1. Scientists have trying to fabricate sensors for detecting ATM1 with a combination of different kinds of conductive elements and semiconducting/insulating substrates.

Table 2 shows a comparison between the performances of the sensors based on different detection techniques. Each of these sensors detecting ATM1 compounds is capable of generating excellent analytical performances. This paper explains the use of sensors formed using CNTs and graphene for the detection of ATM1. Followed by the introduction stating the need for CNTs and graphene-based sensors and their deployment to detect ATM1, Section 2. explains some of the significant kinds of prototypes that are fabricated and utilized for the target application. This section is sub-categorized into two parts based on the type of conductive element used to fabricate the sensors. The third section highlights some of the challenges related to the current sensors and their possible remedies. The conclusion is drawn in the final section of the paper.

## 2. Carbonaceous Sensors for the Detection of Aflatoxin MI Molecules

The consideration of carbon nanostructures for detecting electrochemical sensing applications is significant due to their high sensing area, high aspect ratio and high electrical conductivity. These nanostructures have been able to detect chemical ions in the solutions for a wide range of concentrations. Different detection mechanisms are carried out to determine the changes in the ionic and faradic currents flowing between the electrodes and the tested samples. The changing current values occur due to the corresponding changes in the impedance values due to the specific electrode structures. CNTs and graphene have been utilized to form electrodes that display high selectivity and high sensitivity towards the ionic samples. The deployment of these sensors for food materials has been particularly essential due to their biocompatible nature. While graphene has been a magic material for sensorial operations, other CNT products have also been very efficient as a replacement of the metallic nanowires due to their resistant nature towards a wide range of temperature and humidity and ability to withstand extreme ambiance conditions in terms of heat and cold [89,90]. Both two-electrode and three-electrode systems were equally effective for detecting the ATM1 compound due to the high selectivity induced by these sensors. Electrochemical impedance spectroscopy [91,92] and cyclic voltammetry [93,94] were used to determine the changes in the impedance and current, respectively, due to the corresponding change in the concentrations of the samples.

The doping of these carbon allotrope-based electrodes has positively affected the selectivity and sensitivity towards the aflatoxin molecules. The change in the structural, morphological and electrochemical properties of these electrodes has increased the charge-transfer rate, thus increasing the electrochemical activity on the surface of the electrodes. This treatment is done using a range of active materials such as metallic nanoparticles and polymers that participate in the electrochemical redox reactions. The doping or alteration of the physiochemical nature of the electrodes is done through different techniques such as nanocomposite formation [95], pyrolysis [96], calcination [97] and reduction [98] processes. These treatment methods improve the electron transfer kinetics and charge carrier density at the electrode surface by instigating surface states and facilitating electron transfer [99]. The doped element would also sometimes have multifunctional roles, where a specific area of the electrodes would be activated multiple times as compared to the inactivated ones. This would create mesoporous structures that pave the way for new ionic and faradic reactions, contributing to the change in the output of the devices. The morphological changes can be ascribed to creating new defects on the graphene sheets and sidewalls of CNTs. The decreased amount of amorphous carbon compared to their pristine condition creates small clusters, thus modifying the surface of the particles and increasing their active surface sites. The inhomogeneity caused in the carbon allotropes, although increasing in the charge distribution across the carbon network, results in poor cyclability due to permanent structural changes [100].

The detection phenomenon of ATM1 is based on the ionic bonds formed on the sensing area of the electrodes. The presence of a large aromatic ring of ATM1 helps the sensors to adsorb the compound and separate it for analysis purposes. This helps the researchers to build prototypes that are highly selective towards this particular compound. Although other molecules also consist of aromatic rings, the selectivity generated on the electrodes assists in sensing the aflatoxin molecules. This selective nature can be achieved in multiple ways. It can be done by using aptamers, which act as recognition elements [101]. Initially, aptamers are immobilized on the electrodes via covalent bonding. Then, electrochemical probes are formed with the presence of appropriate signal enhancement elements to induce a charge shift. This causes a change in response during the aptameter-specific recognition and binding of aflatoxin molecules at various levels. Other ways include the presence of nanoparticles such as gold or platinum on the carbon-based electrodes. The presence of these additional nanoparticles modifies the resultant electrocatalytic behavior of the electrodes, thus responding to the aflatoxin molecules when monitored through voltammetry and impedimetric techniques [84]. The selectivity is also achieved through a label-free biosensing mechanism. Here, the carbon-based allotropes form conjugates with other selective nanomaterials and consequently mixes with free antibodies to increase the resultant absorption intensity. Sometimes, solutions such as bovine serum albumin (BSA) are used to increase the sensitivity by blocking the unspecified sites to prevent non-specific adsorption. Finally, the changes in responses of the sensors when treated with aflatoxin antigens are monitored using optical and electrochemiluminescent techniques [83].

When the prototypes are exposed to ATM1 in different concentrations, an adsorption process occurs, the rate of which initially increases and then gradually saturates over time. This equilibrium is attended due to the adsorption of ATM1 on the exterior surface of the adsorbent during the earlier phase of the contact time [85]. After a saturation occurs, the ATM1 ions diffuse from the exterior to the interior surfaces of the adsorbents. Different kinds of sensitive analytical methods are being employed to monitor the ultra-trace levels of ATM1 in milk and other food products. As a result of its strong toxic effects on public health, affordable, portable and efficient methods are devised for screening and detecting the ATM1 levels in the food products. The fabrication and implementation of these sensors have created a podium for further scientists to work on the quality control of the food molecules. Apart from this chosen molecule, other constituents can also be detected with multifunctional sensing systems formulated with carbon-based allotropes.

### 2.1. Carbon Nanotube-Based Sensing Prototypes

Before using graphene as a common carbon-based allotrope for a wide range of sensing applications, CNTs were highly favored due to their attributes. These sensors are particularly beneficial for these kinds of electrochemical sensing due to their sensing area due to their high aspect ratio. Due to the rolling up of graphene sheets into tubes to form CNTs, their electrochemical properties can be compared to the basal planes of the pyrolytic graphite. The cap regions of the CNTs have a higher reactivity due to the higher curve strain in comparison to the sidewall. In retrospect to the defect-free structures of these tubes, their physical and chemical treatments can induce a variety of oxygen-containing groups. This increases the number of binding sites for a particular chemical analyte [99,102]. The high electrical conductivity of CNTs also helps in electrochemical reactions by increasing the electron transfer reactions in both aqueous and non-aqueous solutions. These CNTs have also been used as pastes using different modifiers to form electrodes with high renewability and compatibility.

#### 2.1.1. Multi-Walled Carbon Nanotube (MWCNT)-Based Detection

Interesting research related to the use of CNTs for detecting ATM1 can be seen in the work done by Zhao et al. [103]. The magnetic solid-phase extraction technique was used to develop composites containing PEGylated MWCNTs and metallic magnetic nanoparticles (PEG–MWCNTs–MNP). These prototypes were used to isolate and enrich different aflatoxin compounds such as B1, B2, G1, G2, M1, M2 and others. The responses of the sensors were studied using liquid chromatography and Q-Executive high-resolution mass spectroscopy techniques. The prototypes showed very high sensitivity and selectivity towards the tested molecules. High linearity of coefficient of determination (R^2^) ≥ 0.995 was obtained for a detection range of 0.005–0.050 µg/kg of all the chosen compounds. The quantification range was fixed for a range of 0.015–0.15. The recovery rate was around 81.8–106.4%, along with good repeatability with a range of 2.1–8.5%. The intra-day and inter-day precisions ranged from 2.1 to 8.5% and from 3.9 to 11.7%, respectively. The tested samples were pre-treated with acetonitrile, followed by transferring them for the adsorption process on PEG–MWCNTs–MNP via employing vortex process. The prototypes were capable of detecting thirteen different types of mycotoxins while analyzing twenty milk samples. The ATM1 was detected within a range from 0.026 µg/kg to 0.039 µg/kg.

**Figure 1:**
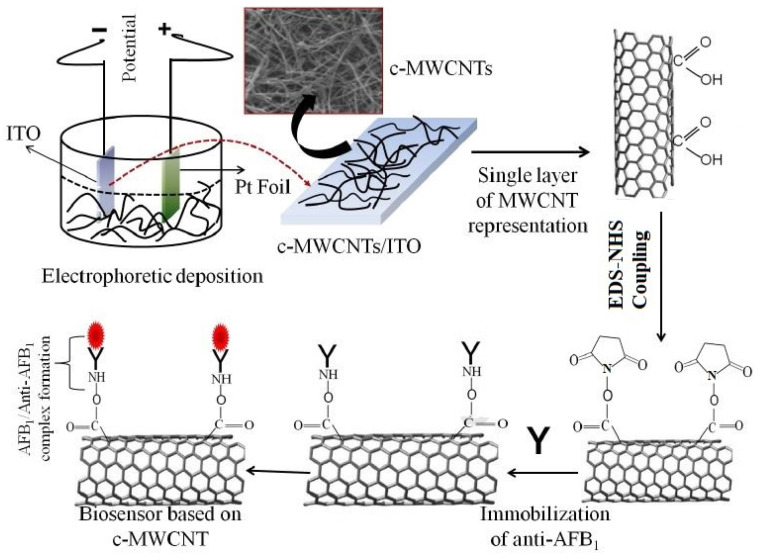
Schematic diagram of the experimental process of detecting AFB1 molecules using carboxyl group functionalized-MWCNTs [104]. Reproduced from Singh, C., Srivastava, S., Ali, M.A., Gupta, T.K., Sumana, G., Srivastava, A., Mathur, R.B. and Malhotra, B.D., 2013. Carboxylated multiwalled carbon nanotubes based biosensor for aflatoxin detection. *Sensors and Actuators B: Chemical*, *185*, pp. 258–264.

As a variant to ATM1, aflatoxin B1 (AFB1) is also detected, a pre-metabolized version of ATM1. For example, Singh et al. [104] showed the use of functionalized MWCNTs to detect aflatoxins. The functionalization of the MWCNTs was done using the carboxyl (–CHO) group. The sensing surfaces of the prototypes were successful in functionalizing with monoclonal AFB1 antibodies to detect these molecules. The functionalization of the MWCNTs was done using a mixture formed with hydrocarbon as a source and ferrocene and toluene as catalysts. The prototypes were formed by electrophoretic deposition of MWCNTs on indium tin oxide (ITO) glass substrates. The DC voltage during the electrophoretic deposition was kept to a constant value. Figure 1 [104] illustrates the experimental process carried out with the MWCNT/ITO-based electrodes. The anode and cathode used for this system were ITO glass and platinum foil, respectively. The electrochemical impedance spectroscopy (EIS) technique was used to detect the changes in the responses of these immunosensors. The immobilization process was done using an anti-AFB_1_ solution, where its amide (–NH_2_) group formed a covalent bond with the –COOH terminal of MWCNTs. The immobilized electrodes were stored at a temperature of 4 °C prior to and after use. The sensors had a high sensitivity of 95.2 µA. mL/ng^−1^.cm^−2^, limit of detection (LOD) of 0.08 ng/mL and linear range of 0.25–1.375 ng/mL. The association constant value of 0.0915 ng mL^−1^ indicated their high affinity towards the detected molecule.

**Figure 2:**
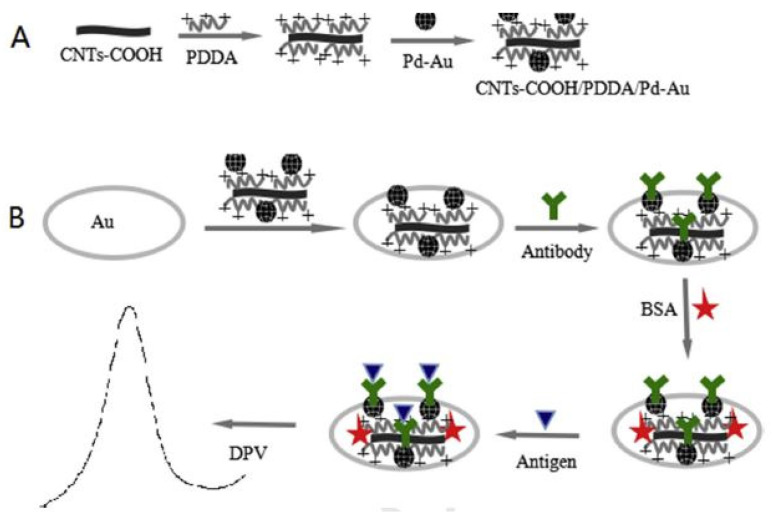
Schematic diagram of the fabrication process of the MWCNTs/PDDA/Pu–Au nanocomposite-based electrochemical immunosensor [105]. (**A**) first part of process, (**B**) second part of process. Reproduced from Zhang, S., Shen, Y., Shen, G., Wang, S., Shen, G. and Yu, R., 2016. Electrochemical immunosensor based on Pd–Au nanoparticles supported on functionalized PDDA–MWCNT nanocomposites for aflatoxin B1 detection. *Analytical biochemistry*, *494*, pp. 10–15.

Another use of MWCNTs for forming nanocomposite-based sensors to detect aflatoxin molecules can be seen in [105]. Here, electrochemical immunosensors for detecting AFB1 molecules were fabricated using palladium (Pd)–gold (Au) nanoparticle-based electrodes that were being supported by poly (diallyl dimethylammonium chloride) (PDDA)/MWCNT-based nanocomposites. Figure 2 [105] shows the schematic illustration of the preparation of an MWCNTs/PDDA/Pd–Au nanocomposite-based electrochemical immunosensor. The Pd–Au nanoparticles were initially synthesized using a dropping and stirring process. This was followed by forming the CNTs–PDDA nanocomposites by treating CNTs with acid and then PDDA aqueous solutions. The resultant suspensions were subjected to centrifugation and sonication processes. The gold electrodes were polished and washed with alumina slurry, followed by distilled water and ethanol. This was followed by dropping samples of CNTs/PDDA/Pd–Au on the surface of the gold electrodes, followed by the evaporation process of the solvent. The experimental samples were prepared using an extraction process, where the samples were shaken, centrifuged, filtered and diluted to obtain the final product. The prototypes showed a high sensitivity for a range from 0.05 ng/mL to 25 ng/mL, along with an LOD of 0.03 ng/mL. The standard deviation was 3σ, where σ is the standard deviation of the blank solution with n = 10. A R^2^ value of 0.9933 was obtained with the peak current in µA decreased with respect to the increase in concentrations of AFB1 samples.

Another interesting work related to using MWCNT-based immunosensors for the detection of aflatoxin molecules can be seen in [106]. Simultaneous detection of AFB1 and zearalenone (ZON) molecules was done using magnetic nanoparticles formulated using amino-modified MWCNTs. The advantages of this proposed work included a convenient and time-saving approach and the fast, efficient and enhanced response of the sensors. The magnetic properties were induced on the –NH_2_-functionalized MWCNTs by filling the nanotubes with Fe_3_O_4_ nanoparticles. This was done by treating the nanotubes with ammonium iron sulfate hexahydrate solutions. The mixture was then optimized in terms of pH values, followed by executing filtration, washing and drying processes. Certain processes such as magnetic solid-phase extraction and high-performance liquid chromatography were used to detect the changes happening in the presence of the two molecules. The influence of other solid-phase extraction parameters such as solution pH, salt addition, temperature, desorption conditions and extraction time was also tested. The sensors showed an excellent response in green analysis, having a score of 89 in analytic eco-scale evaluation. The recovery and relative standard deviation (RSD) ranges of these sensors were 88.8–96% and 2.1–2.8%, respectively. The LOD for AFB1 and ZON molecules were 0.15 ng/g and 0.24 ng/g, respectively, with a R^2^ value of >0.999 for both the molecules. The limit of quantification (LOQ) values for AFB1 and ZON molecules were 0.52 ng/g and 0.83 ng/g, respectively.

Costa et al. [107] showed a similar work regarding the detection of AFB1 molecules using cysteine-modified gold electrode-immobilized MWCNTs. Label-free electrochemical sensors were fabricated for the detection of carcinogenic AFB1 molecules in pictogram levels. The presence of the MWCNTs assisted in the enhancement of the electrical properties in terms of sensitivity and working range. The gold electrodes of the sensors were initially modified using a self-assembled cysteine layer, followed by covalent bonding of carboxyl-functionalized MWCNTs to the self-assembled layer. With an initial polish using α-Al_2_O_3_ and an ultrasonication bath, the modification was done to create the self-assembled layer. Drop-casting of the carbonyl-MWCNT-diluted solutions was done on the electrodes, followed by an incubation bath for 50 min at a temperature of 25 °C. Finally, tethering of antibodies was done against AFB1 molecules. This was carried out by drop-casting the EDC:NHS diluted solution at a ratio of 1:1 over the activated platform. The tested AFB1 concentrations included 0.1, 1.0, 3.0, 6.0, 9.0, 11.0, 15.0 and 20.0 pg/g. The charge-transfer resistance changed for a range between 6.97 kΩ and 84.33 kΩ for the lowest and highest concentrations, respectively. The LOD of these sensors was 0.79 pg/g, while the linear response was from 0.1 pg/g to 20 pg/g. Reproducibility in the results of around 5% was obtained for these portable, label-free sensors.

One of the recent works elucidating the use of MWCNTs for the detection of aflatoxins in milk can be seen in [108]. Polyethyleneimine (PEI) was used as a solid-phase extraction adsorbent to functionalize the magnetic CNTs. Magnetic substrates and epoxy-containing silane agents as linkers were employed for the modification of polyethyleneimine. MWCNTs were used as the conductive material, which was functionalized with iron oxide (Fe_3_O_4_) molecules to induce magnetic properties in the CNTs. This was followed by two more modifications with PEI and glymo to obtain a final product of glymo@Fe_3_O_4_MWCNTs. The analysis was carried out using magnetic adsorbents integrated with reverse phase and anion exchange interaction sites. The capability of these sensors was validated through different analytic methods in terms of linear range, absolute recovery, matrix effect and precision. The experimental process consisted of testing ten different mycotoxins in milk, having the range of adsorption capacities between 4.9 mg/g and 10.2 mg/g. The adsorption and desorption processes were completed within 3 min and 2 min, respectively. The recovery rates increased with the increase in the amount of MWCNTs but were consistent with the variation in the adsorption capacities. The correlation coefficients obtained ranged between 0.9108 and 0.9981. The standard deviation and LOD ranges were 2.4% to 6.5% and 0.003 µg/kg to 0.334 µg/kg, respectively. The sensors also had a high recovery range from 88.3% to 103.5%.

#### 2.1.2. Single-Walled Carbon Nanotube (SWCNT)-Based Detection

**Figure 3:**
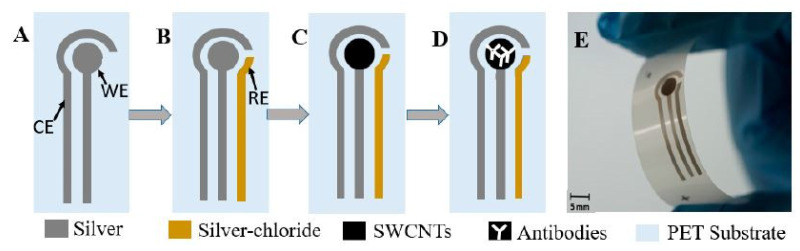
Schematic diagram of the fabrication process of SWCNT/PET-based biosensors [109]. (**A**) printing working electrode (WE) and counter electrode (CE), (**B**) printing WE with AgCl by alignment, (**C**) spray depositing single-walled carbon nanotubes (SWCNTs), (**D**) immobilization of antibody, and (**E**) final biosensor. Reproduced from Abera, B.D., Falco, A., Ibba, P., Cantarella, G., Petti, L. and Lugli, P., 2019. Development of flexible dispense-printed electrochemical immunosensor for aflatoxin M1 detection in milk. *Sensors*, *19*(18), p. 3912.

Like MWCNTs, SWCNTs have also been used to detect ATM1 compounds in milk [109]. Flexible prototypes having dispense-printed electrodes were formed where functionalized SWCNTs were coated with antibodies to increase their selectivity and sensitivity. The advantages of these sensors were low fabrication cost, easy handling and a high level of customization. Figure 3 [109] shows the schematic diagram of the fabrication process of these functionalized SWCNT-based biosensors. These prototypes were formed on PET substrates having a thickness of 125 microns. The printing process was followed by curing them at 120 °C for 15 min to harden the ink.

The final step included the spray-coating of the SWCNTs on top of the electrodes via a spray deposition unit and a shadow mask. The cyclic voltammetry (CV) technique was associated with the prototypes to detect the output with respect to the corresponding ATM1 compounds. The working principle is based on a three-electrode system, with silver paste printed with working and counter electrodes and Ag/AgCl being the reference electrodes. The immobilization process was done using the drop-casting technique, where secondary antibody solutions were used to treat the working electrode. Prior to the sample testing, the milk was defatted by centrifugation process at a speed, duration and temperature of 6000 rpm, 15 min and 4 °C, respectively. The LOD of the sensors was 0.025 µg/L. Both the functionalized and un-functionalized electrodes had the same working range between 0.01 µg/L and 1 µg/L.

One of the interesting works highlighting the conjugation of SWCNTs with nanoparticles can be shown in [110], where the electrodes were formed using SWCNTs and gold nanoparticles (Au NPs) in chitosan. The immobilization of the sensing area was done using tyrosinase enzymes that showed a reversible behavior. The SWCNTs were treated with different solutions such as nitric acid, sulphuric acid and chitosan to form homogeneous solutions. The biosensitive part of the prototypes was prepared by treating them with BSA phosphate buffer solutions, distilled water and glutaraldehyde solution. Finally, the sensing surface of the screen-printed electrodes was modified using Au NPs that were obtained as pink wine-red solutions. The kinetic studies of these sensors were done at a concentration of 1 × 10^−3^ M. The working range and LOD of the sensors were 1 × 11^−11^ M–1 × 10^−6^ M and 5 × 10^−12^ M, respectively. The sensors showed a two-parameter mismatch inhibition for the composites developed with CNTs and Au NPs. The maximum level of inhabitation by ATM1 on these sensors was 78 ± 1.0%. When the performance of these sensors was compared to the ones developed using tyrosinase-functionalized screen-printed graphene oxide sensors, it was seen that the ones formed using CNTs/Au NPs had a higher sensitivity and correlation coefficient.

The work done by Gan et al. [85] can be exemplified to show the use of SWCNT-based composites for developing electrochemiluminescent immunoassays (ECLIA) for the detection of ATM1 in milk. The working principle of these ELCIA-based sensors was based on two parts, namely the extraction and detection processes. The extraction process was carried out using magnetic graphene, while the detection part was done by nanocomposites formed by mixing antibody-labeled cadmium telluride (CdTe) quantum dots and SWCNTs. The CdTe quantum dots were used as signal tags, which were attached to the primary ATM1 antibodies in the form of nanocomposites. The immobilization of GO was done with Fe_3_O_4_ nanoparticles to develop the magnetic nanocomposites for absorbing the ATM1 compounds. In order to form the conjugates, stirring and ultrasonication processes were deployed after the reaction of SWCNTs with dimethylformamide (DMF) and polydiallyldimethylammonium chloride (PDDA) solutions, respectively. CdTe quantum dots having free surface –COOH groups were used to form dispersions, followed by reacting them with SWCNTs–PDDA solutions to obtain the final solution having a 1:1 concentration ratio. The labeling of the antibodies was done using a centrifugation process with a speed and duration of 5000 rpm and 10 min, respectively. The adsorption process was done for pH values ranging between 3.0 and 8.0, where 95% of the equilibrium process was achieved within 10 min. The sandwich ECLA process was employed for the detection of ATM1 in milk samples. The linear range of the sensors was from 1 pg/mL to 1 × 10^5^ pg/mL, along with a LOD of 0.3 pg/mL. The testing was done for ten milk samples, the results of which proved to be more effective than the standard ELISA method. Table 3 shows a comparison between the performances of the CNT-based sensors to detect ATM1 and AFB1 molecules. It is seen that the performances of the prototypes are largely dependent on the type of processed materials being associated with these CNTs.

### 2.2. Graphene-Based Sensing Prototypes

Graphene has been one of the most widely-used carbon allotropes for developing flexible sensors for a wide range of applications. Due to the enhanced electrical, mechanical and thermal characteristics, graphene in its pure, oxide and composite forms has achieved the required performances. Biomedical sensing applications have also been used to detect biomolecules [111,112,113] to their high surface-to-volume ratio, excellent electrical conductivity and tunable optical properties. This helps the sensors to achieve high sensitivity, low LOD and detection without any chemical mediators [111]. Apart from this, the advantage of the biocompatibility of graphene has led many research groups and industries to develop wearable graphene-based sensors for ubiquitous applications.

#### 2.2.1. Graphene Oxide-Based Detection

**Figure 4:**
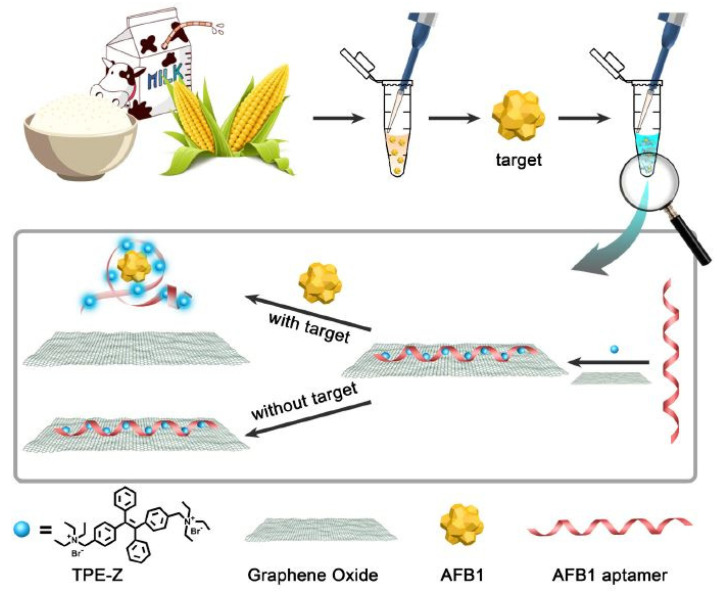
The working mechanism of the TPE-Z/AFB1 aptamer/GO-based devices for the detection of AFB1 molecules in different types of food samples [112]. Reproduced from Jia, Y., Wu, F., Liu, P., Zhou, G., Yu, B., Lou, X. and Xia, F., 2019. A label-free fluorescent aptasensor for the detection of Aflatoxin B1 in food samples using AIEgens and graphene oxide. *Talanta*, *198*, pp. 71–77.

Jia et al. [112] showed the fabrication and implementation of label-free fluorescent aptasensors to detect AFB1 molecules. The constituents of these sensors included the quaternization of tetra-phenylethene salt (TPE-Z), GO and AFB1 aptamer. The primary advantage of this work includes the single-step operation process compared to the other aptamer used for detecting AFB1 molecules with GO-based sensors. Figure 4 [112] represents the working mechanism of these label-free aptasensors that were used for the detection of AFB1 molecules. The operating principle of these sensors was based on the conformational switch of the AFB1 aptamer from the single-stranded structure to the aptamer complex. This process leads the GO to release the TPE-Z/AFB1 aptamer from its surface. The fluorescence intensity was recorded at a wavelength of 480 nm, having an excitation at 340 nm. The sensitivity values were measured three times for each concentration. The LOD of these devices was 0.25 ng/mL. These sensors showed the capability to selectively detect aflatoxins in certain foods such as milk, corn and rice. Before using these food samples, extraction was done using methanol–water, centrifugation and filtration processes. Finally, the supernatants were taken for analysis purposes. The sensors could detect AFB1 molecules from all three types of food, with the recovery rate ranging between 91% and 95%.

Guo et al. [113] also showed the use of GO to develop an aptasensor to detect ATM1 in milk products. The GO particles also quenched on the fluorescence principle, where it was done on an aptamer labeled carboxyfluorescein while protecting it from nuclease cleavage. The presence of ATM1 formed a complex, which resulted in its detachment from the surface of the GO and causing aptamer cleavage by DNase I. The GO binding with the ss-DNA via π-stacking interactions helped with the high distance-dependent fluorescence quenching performance. This was followed by the detachment of target AFM1, leading to a new cycle. The testing was done with infant milk powder samples. The samples were spiked with AFM1 molecules at four concentrations of 0, 1.5, 2.5 and 5 µg/kg.

After the weight of the samples was measured carefully, the extraction process was carried out, and the supernatant was concentrated under a nitrogen stream. Finally, the residues were mixed with methanol solution and used for fluorescence signal amplification experiments. The linear response and LOD of the sensors were 0.2–10 µg/kg and 0.05 µg/kg, respectively. The recovery rate for the chosen spiked concentrations was between 98% and 126%, with RSD values of 0.06 and 0.42. The prototypes also showed high sensitivity and selectivity towards the AFM1 molecule without the presence of any interference. Another research by Zhang et al. [114] showed the use of fluorescence quenching immunoassay and graphene-based composites. The assays were formed using a monoclonal antibody (mAb)-functionalized Fe_3_O_4_-decorated GO that acted as a capture probe and energy acceptor. These assays were combined with tetramethylrhodamine cadaverine-labeled AFB1 molecules. The advantages of these sensors included their single-step preparation and detection process, low quantitative detection limit, low cost of the sensors, precise quantitative analysis and the entire process being completed within 10 min. The magnetic rGO was formed by processing different solutions such as iron chloride and ferric chloride, forming dispersions with GO solutions. These dispersions were processed using techniques of ultrasonication and centrifugation to obtain the final product. The sensors were tested with ATM1 concentrations ranging from 0.01 ng/L and 2 ng/L to obtain recovery rates ranging between 94.4% and 104.5%. High linearity of R^2^= 0.999 was also obtained with these sensors’ performance compared to a commercialized enzyme-linked immunosorbent assay (ELISA) kit. With a high reproducibility of the results, the values of coefficients of variation were under 6.2%. The visual and quantitative LOD were 50 ng/L and 3.8 ng/L, respectively.

An example showing the use of both graphene and crystalline quantum dots for forming fluorescence assays for the detection of aflatoxin molecules can be seen in the work done by Lu et al. [115]. GO was linked with CdTe quantum dots via ligand exchange as a quenching process to determine the performance of the fluorescence-based devices. The GO was synthesized using Hummers’ method, followed by mixing it with hydrogen peroxide to form GO suspensions. The CdTe quantum dots were formed by processing trisodium citrate dehydrate and cadmium chloride solutions using stirring, followed by heating them inside a Teflon-lined stainless autoclave. The temperature and duration were maintained at 180 °C and 35 min, respectively. Finally, the samples were washed and centrifuged to obtain the pigmented layers and remove the residual chemicals. Finally, the individual conductive materials were conjugated with the aptamer and centrifuged again, both the composites being required to detect AFB1 molecules. The prototypes showed good sensitivity and selectivity and had a wide dynamic range of 3.2–320 nM. While the LOD of the sensors was 1 mM, they achieved high linearity with R^2^ = 0.998. They showed high selectivity towards the aflatoxin molecules in the presence of other molecules such as fumonisin B1, ochratoxin A, zearalenone and deoxynivalenol. The response of these sensors in terms of fluorescence intensity with the corresponding enhancement of the AFB1 concentration ranged from 1.6 nM to 160 µM.

**Figure 5:**
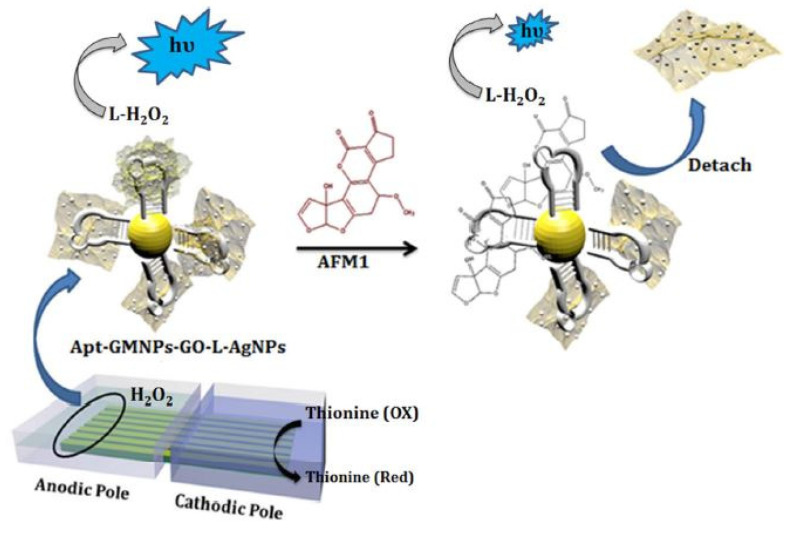
Representation of the immobilization process of the ATM1-based antibody with the luminol-functionalized Ag NP decorated GO [116]. Reproduced from Khoshfetrat, S.M., Bagheri, H. and Mehrgardi, M.A., 2018. Visual electrochemiluminescence biosensing of aflatoxin M1 based on luminol-functionalized, silver nanoparticle-decorated graphene oxide. *Biosensors and Bioelectronics*, *100*, pp. 382–388.

Apart from these quantum dots and nanosheets, graphene oxide (GO) has been largely preferred to form the prototypes to detect aflatoxin molecules. The primary reason behind this is its ability to form homogeneous dispersions, necessary for forming these electrochemical sensors. One such work can be highlighted in [116], where the prototypes were formed by functionalization of luminol on Ag NP-decorated GO. Bipolar electrode arrays were formed that operated on the visual ECLIA biosensing technique. The Au NPs were coated with magnetic Fe_3_O_4_ nanoparticles before their immobilization with thiolated ATM1 aptamer. Figure 5 [116] shows the schematic diagram of the immobilization of the antibody with these GO nanocomposite-based sensors. The operating mechanism of these sensors depended on the π–π interactions of the nanocomposites with the unpaired bases of the immobilized aptamer. The composites consisting of GO, luminol and Ag NPs were synthesized using the one-pot method. Before the experimental process, the anodic poles were modified with the nanocomposites on the gold bipolar electrode arrays. The Ag NPs assisted in the catalysis of the ECL process on the sensing surface of the prototypes. The optimal conditions were achieved using a face-centered central composite design, where the aptasensors obtained a linear response for a dynamic range between 5 ng/mL and 150 ng/mL. This range increased to 10–200 ng/mL when smartphones were embedded with the sensors for using them for point-to-point services. The LOD of the sensors without and with the smartphones was 0.01 ng/mL and 0.05 ng/mL, respectively. A reliability reproducibility was also obtained with an RSD of 2.3%.

Another example showing the use of GO can be shown in [117], where impedimetric sensors were formed using single-stranded Herring sperm DNA (ss-HSDNA) and reduced graphene oxide (rGO) for the detection of AFB1 molecules. These aerogel-labeled prototypes were quantified on rotating disk electrodes, where the ss-HSDNA and rGO were conjugated to operate with the CV technique. The rGO aerogel was formed using the Hummers and Offman method with a slight change in the modification of the use of Milli-Q water for 30 min. The GO suspensions were then treated with hydrazine for reducing them, followed by soaking and drying them to obtain the final product. The modification of the electrodes was done by drop-casting on glassy carbon rotating disk electrodes having a diameter of 3.0 ± 1.0 mm. These glassy substrates were then polished with alumina and washed with purified water before their experimental uses. The consideration of the presence of FcCH_2_OH as the redox mediator was done to detect the hydrodynamic diffusion effect of the ss-HSDNA/rGO-based devices. Two techniques, namely electro-redox mediators and the hydrodynamic effect, were considered to obtain three different charge values of 825 mA, 615 mA and 550 mA. These anodic current values were obtained at a scan rate of 50 mV/s. The rotating speed of the disk electrodes ranged from 500 rpm to 4000 rpm. The linear range of the sensors was from 1 × 10^−1^ g/mL to 7 × 10^−8^ g/mL, with an LOD of 0.04 ng/mL.

Jiang et al. [118] showed an interesting work where the graphene-based sensors were used to detect nine mycotoxins in milk. The sensors were formed using rGO and gold nanoparticles. Two different processes, namely solid-phase extraction coupling of ultra-high-performance liquid chromatography–tandem mass spectrometry, were used as the detection mechanisms. The rGO/gold nanoparticles composite was formed using stirring, centrifugation and annealing processes. Before their use, the synthesized composites were stored at a temperature of −20 °C and cryodesiccated. The experiments were conducted with milk to determine these nine types of mycotoxins that included the AFB1 and ATM1 compounds. The milk products were spiked with each of these nine compounds for 1, 20 and 100 ng/mL concentrations. A total of sixty milk samples were tested during the entire experimental process. The testing samples were prepared by using three different types of solutions. The loading and washing solutions included 2% acetonitrile/formic acid and 5% methanol in water, respectively. These graphene-based sensors showed excellent analytical response with a high sensitivity of 0.02–0.18 ng/mL, recovery of 70.2–111.2% and a precision of 2–14.9%. The linearity of the sensors was satisfactory with R^2^ ≥ 0.992. The LOD and LOQ values of the sensors were 3 and 10, respectively.

**Figure 6:**
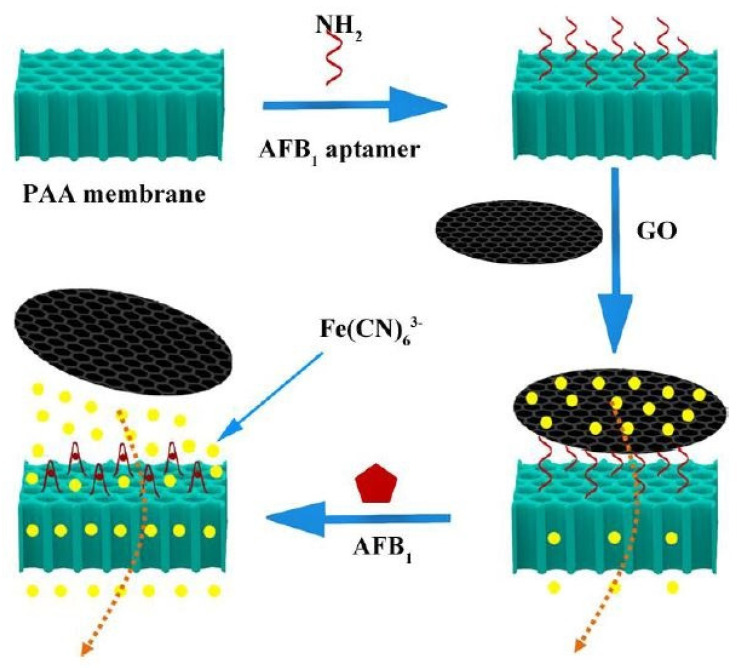
Illustration of the working mechanism of the GO-modified PPA membranes for the detection of AFB1 molecules [119]. Reproduced from Mo, R., He, L., Yan, X., Su, T., Zhou, C., Wang, Z., Hong, P., Sun, S. and Li, C., 2018. A novel aflatoxin B1 biosensor based on a porous anodized alumina membrane modified with graphene oxide and an aflatoxin B1 aptamer. *Electrochemistry Communications*, *95*, pp. 9–13.

Mo et al. [119] worked on the development and implementation of AFB1 biosensors based on porous anodized alumina (PAA) membranes. These membranes were modified using GO and AFB1 aptamers. Figure 6 [119] shows the schematic diagram of the working mechanism of these biosensors. After being synthesized using Hummers’ method, the GO was processed using various chemicals to form GO hydrosol. These hydrosols were centrifuged, washed, dialyzed and finally stored. The modification of the PAA membranes was done by treating them with 5% 3-aminopropoyltrimethoxysilane solution for 12 h.

Finally, they were again washed and dried at 110 °C for an hour to form a silane layer. This was followed by the immobilization process, which took place inside a customized electrocatalytic cell. The GO was attached to the aptamer via π–π stacking, which led to increased negative charge of the nanochannels. This change in charge density and steric hindrance leads to the flux of ferricyanide ions through the nanochannels, thus increasing overall current. These devices were selective towards three molecules, namely ochratoxin, aflatoxin G1 and AFB1. They were tested for a concentration of 10 ng/mL for AFB1 by immersing the sensors inside the samples for 90 min and analyzing the current change with respect to time. The experimental results showed that the current increased properly with respect to the concentration of AFB1 molecules. The sensors had a linear range of 1–20 ng/mL and a LOD of 0.13 ng/mL.

#### 2.2.2. Other Types of Graphene Nanostructure-Based Detection

For the detection of ATM1, one of the interesting examples can be given by highlighting the work done by Shadjou et al. [120]. Graphene quantum dots (GQDs) were employed in conjugation with silver nanoparticles (Ag NPs) to form the electrochemical sensors. These sensors also contained a member of cyclic oligosaccharides, α-cyclodextrin, which increased the sensitivity of these multi-layered films. These nanocomposite-based sensors were formed on glassy carbon electrodes due to their high surface area available for detection purposes. The GQDs were formed by dispersing carbonized products into alkaline solutions by pyrolyzing citric acid. The samples were then treated with sodium hydroxide and were stirred to obtain the final result. These GQDs were electrodeposited on the surface of the glassy carbon electrodes with a potential and scan rate of 0–1 V and 100 mV/s, respectively. The CV technique was conducted during the electrodeposition process to analyze, optimize and obtain thin-film electrodes. These samples were then used for the electro-polymerization of α-CD using the CV technique again with a voltage sweep between −1 V and 1 V and a scan rate of 100 mV/s for ten cycles. The final step included the electrodeposition of Ag NPs on the surface of the electrodes using similar parameters as in the earlier step. The milk samples used for experimental purposes consisted of local and pasteurized milk. The linear range of these sensors was from 0.015 mM to 25 mM, with an LOQ of 2 µM. The capability of these sensors was validated by analyzing their performances with respect to solution pH, potential scan rate, reproducibility and stability.

Another work related to the use of GQDs can be seen in [121], where electroluminescence (ECL) aptasensors were developed for the detection of AFB1 molecules. The GQDs were used to form nanocomposites that consisted of gold nanorods, poly (indole-6-carboxylic acid) and flower-gold. The ECL aptasensors were formed by using the potentiostatic method on glassy carbon electrodes. The final nanocomposite solution was incubated with different concentrations of AFB1 molecules at a temperature of 37 °C for 80 min. The characterization of the nanocomposites was done using the EIS technique, while the experimental process included the study of ECL intensity. The nanocomposites having a core–shell structure had certain advantages such as high electrical conductivity and superior luminescent performance. The immobilization of gold nanorods was done with an AFB1 aptamer chain to enhance the overall selectivity and sensitivity. AFB1 standard solutions were formed by adding 1 nM, 2 nM and 5 nM of AFB1 molecules to the actual samples. The recovery values of these sensors were 97.1–111.8%, which was quite close to the recovery values of the standardized high-performance liquid chromatography fluorescence (HPLC-FL) (90.7–114.7%) process. The prototypes had a wide dynamic range from 0.01 to 100 ng/mL with an LOD of 0.00375 ng/mL. Other attributes of the sensors were high stability and reproducibility of the responses, high accuracy and high reliability for real samples analysis.

Another research showing the use of graphene nanostructures for developing flexible prototypes for the detection of aflatoxin molecules can be seen in [122]. The sensors were formed with graphene nanosheets on ITO-coated substrates. The advantages of these sensors are their rapid fabrication process, simple operating principle and low cost. The ITO coating was done on glass substrates with dimensions of 20 mm × 10 mm × 1.1 m. Before and after the coating process, they were cleaned via the sonication process and dried using nitrogen gas. This was followed by electrochemical deposition of rGO nanosheets, followed by electrochemical deposition of gold nanodots using aqueous solutions. The potentials used during the first and second electrochemical deposition processes were −1.6 V and −0.9 V, respectively. The next step included the immobilization of these prototypes with the AFB1 antibody with the self-assembly technique. The duration and temperature were fixed at 6 h and 4 °C, respectively. The CV technique was used as the detection technique to test the food samples spiked with AFB1 molecules. The spiking was done with three different concentrations, namely 10 ng/mL, 50 ng/mL and 100 ng/mL. These electrochemical sensors were used to detect AFB1 molecules with a LOD of 6.9 pg/mL. The sensors retained around 96% of their original response while experimenting with 50 ng/mL concentration for ten days.

Another variation in the graphene structure can be shown in Tezeji et al. [123]. β-cyclodextrin was developed using a facile one-pot green synthesis method and was subsequently decorated using graphene nanohybrids. Some of the advantages of these sensors were high specific surface area and high supramolecular recognition and enrichment capability. The functionalization of the β-cyclodextrin was carried out using hydrothermal reaction and reduction processes. After GO was synthesized from graphene nanosheets using the modified Hummers method, they were self-assembled onto β-cyclodextrin. The one-pot hydrothermal process included GO dispersions on β-CD solutions, followed by ultrasonication for 15 min. Finally, the samples were heated, washed with deionized water and frozen at −50 °C to form porous structures. The sensors could detect different types of aflatoxin molecules such as B1, B2, G1 and G2. The LOD and linear range of these sensors were 7.5–30 ng/kg and 25–100 ng/kg, respectively. The LOQ according to the signal-to-noise ratio was 10. The effects of other parameters such as pH, adsorbent amount, sample loading flow rate, ionic strength and reusability of the sensors were also studied. The sensors also showed an excellent recovery rate with a range from 90.5 to 105%, with high accuracy for three concentration levels of 250 ng/kg, 1000 ng/kg and 10,000 ng/kg. The relative standard deviation was less than 6.1% when the testing was done five times in a single day with an interval of one hour. Table 4 showcases a comparative study for the performances of the graphene-based sensors for the detection of ATM1 and AFB1 compounds.

## 3. Results

Although a substantial amount of work has been done in detecting aflatoxin biomolecules in milk and other food products, there are still some bottlenecks that need to be addressed. The formation of homogenous dispersions using CNTs is one issue that remains unsolved. Although surfactants have tackled this problem to a certain degree, these compounds generally affect the mechanical integrity of the nanotubes, which eventually affects the results. The utilization of these prototypes as point-of-care (POC) devices should be further encouraged to obtain a quick and efficient response regarding the concentration of ATM1 and AFB1 compounds in milk and other food products. In order to generate POC devices, three factors need to be taken into consideration [124,125,126,127]. These factors include the use of disposable sensors, amplification of the sensed response via embedded circuitry and the use of open-source software and hardware as the associated electronics. Although the first factor would increase overall cost, it would assist in maintaining a high sensitivity towards the target analyte. The second factor would help in two ways, namely eliminating the unwanted noise and presenting the sensed data in a most easily comprehensible way. The essentiality in using the open-source will guarantee the deployment of standardized procedures used by people who faced a similar situation. It will also help to obtain constructive feedback to improve the quality of the POCs. In the era of 3D printers and laser cutters, open-source hardware is easily available and customizable, assisting in commercializing the sensing systems. Two of the major steps that need to be implemented to obtain a POC device are the fabrication of a lab-on-a-chip sensing system and the replacement of commercially available tools with self-made solutions via open-source resources. An optimization process should be carried out to determine the ideal sample concentration to achieve better responses in terms of the analytical performances of the sensors.

The signal processing part of the electronic circuitry should include microcontroller-based platforms such as Arduino or Raspberry Pi to develop low-cost devices that are easily available to the users. Similarly, the readout part should have simple digital scales for users who have little or no prior experience in electrical engineering. Due to the requirement of microelectronic and cleanroom standard processes, the circuit boards can use nanomaterials and polymers as a replacement for silicon technology. This will not only increase the electrical conductivity and thermal stability but will also increase the biodegradability and biocompatibility of the sensors. The fabrication process to form the POC circuitry can also be altered to form fully integrated fast, portable, low-cost and easy-to-use systems with high sensitivity and specificity [128]. They will help to detect in controlled environments but can be used by non-expert people to perform a long-term study in the change in the concentrations over the due course of time. Ferromagnetic materials other than Fe_3_O_4_ particles should be considered for magnetizing the prototypes. These particles are prone to oxidation in the air due to their high surface chemical activity on their surface [129]. The consideration of forming sensor arrays to detect different types of aflatoxin compounds can also be done in order to reduce the sensor cost and detection time. The POC devices can also be embedded with wireless communication protocols so that the precise concentration of the aflatoxin compounds in milk and other food products can be displayed in local grocery stores and supermarkets. This will assist consumers in deciding if the milk should be bought or not. A dynamic thresholding value [130] can be set so that the change in the concentrations of aflatoxin compounds with respect to certain parameters such as days, type of milk, storage temperature, storage material and others can be monitored. With respect to the detection techniques used in conjugation with these sensors, optical sensing techniques work better in terms of faster response, higher spatial resolution and lower detection limits. This technique should be further employed to detect ATM1 in milk [131] to help these carbon allotropes-based sensors achieve better performances.

## 4. Conclusions and Future Perspectives

The paper highlights the use of CNTs and graphene to develop sensors that have successfully detected ATM1 and AFB1 compounds at varying concentrations. The advantages of these types of sensors include high biocompatibility, easy operating principles and quick fabrication and detection processes. These nanomaterials have been used in both pure and composite forms, in which graphene has been considered in certain shapes such as quantum dots, nanosheets and nanoparticles. The conjugation of these carbon-based allotropes has been done with other nanomaterials such as crystalline compounds and conductive metallic nanoparticles to increase the sensitivity of the sensing area. The selectivity of these prototypes has been increased by attaching primary antibodies so that they show enhanced performances even in the presence of other interfering molecules. The magnetic properties of these sensors have also been altered by treating them with certain ferromagnetic materials. The availability of the prototypes mentioned above for the detection of aflatoxin compounds has provided a strong podium in microelectronics, which can help future researchers develop sensors for the detection of different kinds of biomolecules present in food products.

Further work can be done on enhancing the fabrication and implementation sides of these sensors. In order to develop these sensors, low-cost 3D printed sensors can be developed via an additive manufacturing process. Customized printers should be designed for quick roll-to-roll production of these sensors. Since the addition of a selective layer creates an issue of reusability, the fabrication part should include the processing nanomaterials that form the selective layer. The materials that are chosen to form the selective layer should be more biodegradable and biocompatible material as a replacement to metallic nanomaterials. In terms of detection mechanisms, certain processes such as chromatography and optical techniques should be encouraged ahead of impedance spectroscopic techniques. This will not only help in conducting the experiments with low sample volumes and obtain a precise detection but will also aid in avoiding certain circumstances such as reducing the ambient noise and the minimization of theoretical simulations and complex data analysis for quantification [132]. The single-use sensors can also be motivated in order to avoid sensitivity drift problems and storage issues. The further focus should be given to food packaging industries to integrate these sensors with milk bottles and other food products. The substrates of the prototypes can be replaced with bioplastics in order to make the sensors biocompatible and increase their overall thermal tolerance. The sensors would help to obtain real-time data with regards to the change in the concentration of aflatoxin levels with the help of color-coded statuary warning charts. Research projects should be proposed with a collaboration between the academic groups and food packaging industries so that the sensors fabricated in a controlled laboratory environment can be simultaneously tested in real-time situations. Thorough scrutiny should be done on the expiry and consumable dates of the dairy products so that a maximum number of consumers can avoid the effects caused by the excess amount of aflatoxins in the body. The milk obtained from the dairy cows should be pasteurized properly before packaging, storing and consumption. This is because the food habits of some of the dairy cows contain mycotoxins, which are metalized into carcinogenic ATM1 and are subsequently eliminated through milk [133]. In a broader sense, a classification should be done on the quality of milk available in local stores and supermarkets on the basis of the chemical compounds present in them and the health hazards they can cause when increased beyond a certain threshold. This should also be reflected on the expiry dates so that consumers can have a safe range for their consumption plan. The electronics embedded with the sensors can consist of radio-frequency identification (RFID) tags or other flexible wireless protocols that can immediately send the data within specific ranges. This will be helpful to keep a regular check on the concentration of the aflatoxin, especially in cases of the changes happening in storage and the environment.

## Figures and Tables

**Table 1:** Comparison between CNTs and graphene based on certain physicochemical properties [78].

Physiochemical Properties	Graphene	Carbon Nanotubes
Shape	Planar: monolayer or multi-layer	Cylindrical (SWCNTs, DWCNTs, MWCNTs)
Dimensions	Thickness: 0.34–100 nm Lateral size: 0.3–10 microns	SWCNT length: 10 nm to 1 cm, diameter: 0.4 to 3 nm MWCNT length: 10 nm to few microns, diameter: 2 to 200 nm
Surface	Up to 275 m^2^/g, decreases with an increase in the number of layers. Varies with functionalization or coating. Varies in chemical nature, type, density and conformation.	SWCNTs (>1000 m^2^/g), MWCNTs (100–500 m^2^/g) Varies with functionalization or coating. Varies with chemical nature, type, density and confirmation.
Elasticity/stiffness	Young’s modulus: 1100 GPa Capable of bending and rippling Stiffness increases with the number of layers	SWCNTs: 1 to 5 TPa, capable of bending MWCNTs: 0.2 to 0.95 TPa
Colloidal stability	Dispersion: Graphene oxide in water Aggregation: Stacking	Dispersion: Oxidized CNTs in water Aggregation: Bundling and tangling
Durability	Enzymatic degradation by defects in the plan	Enzymatic degradation by unzipping and decrease in length and diameter
Impurities	Varies after the manufacturing process, mainly graphite and chemical residues after processing.	Varies after the manufacturing process, metal catalysts (Fe, Co, Ni, Cr, Cu, Zn), carbon nanoparticles, amorphous carbon

**Table 2:** Comparison between the performances of the sensing prototypes based on different detection techniques.

Sensor Materials	Detection Technique	Detection Analyte	Analytical Performance	Ref.
Graphene nanoribbons	Dynamic light scattering	AFB1	Linear range: 0.5–20 ng/mL LOD: 0.16 ng/mL	[83]
Graphene oxide, Au NPs, PEDOT	Impedance spectroscopy	AFB1	Linear range: 0.5–20 ng/mL, 20–60 ng/mL LOD: 0.109 ng/mL	[84]
Fe_3_O_4_, GO, CdTe quantum dots, CNTs	Electrochemiluminescence	ATM1	Linear range: 1.0–1.0 × 10^5^ pg/mL LOD: 0.3 pg/mL	[85]
rGO, polyaniline, MoS_2_, glassy carbon electrode, Au NPs	Differential pulse voltammetry	AFB1	Linear range: 0.01–1.0 fg/mL LOD: 1.0 fg/mL	[86]
Au NPs, carboxymethyl-dextran	Cyclic voltammetry	AFB1	Linear range: R^2^ >0.99 LOD: 3.3	[87]
Au NPs	Thin layer chromatography	ATM1	Linear range: 0–80 ng/L LOD: 350 ng/L	[88]

**Table 3:** Comparison of the performances of the CNT-based sensors for the detection of ATM1 and AFB1 compounds.

Processed Materials	Fabrication Technique	Detection Analyte	Linear Range	Limit of Detection	Ref.
Fe_3_O_4_, GO, CdTe quantum dots, CNTs	In situ chemical co-precipitation, ultrasonication	ATM1	1.0 1.0 × 10^5^ pg/mL	0.3 pg/mL	[85]
PEG, MWCNTs, magnetic nanoparticles	Centrifugation, stirring	ATM1	R^2^ ≥ 0.995	0.005–0.050 μg/kg	[103]
Carboxyl-functionalized MWCNTs, ITO, glass substrate	CVD, electrophoretic deposition	ATM1	0.25–1.375 ng mL^−1^	0.08 ng mL^−1^	[104]
PDDA–MWCNTs, Pd Au NPs, Pd NPs	Centrifugation, stirring	AFB1	0.05–25 ng/L	0.03 ng mL^−1^	[105]
Fe_3_O_4_, NH_2_-MWCNTs	Centrifugation, stirring	AFB1	1–100 ng/g	0.15 ng/g	[106]
Carboxyl-functionalized CNTs, anti-AFB1, cysteine	Self-assembly, centrifugation	AFB1	0.1–20 pg/g	0.78 pg/g	[107]
Fe_3_O_4_, MWCNTs, PEI	Centrifugation, stirring	AFB1	R^2^ = 0.9982–0.9997	0.003 μg/kg^−1^–0.442 μg kg−^1^	[108]
SWCNTs, mAb, PET	Screen printing	ATM1	-	0.02 µg/L	[109]
CNTs, graphene oxide, Au NPs, tyrosinase	Centrifugation, stirring	ATM1	-	5 × 10^–12^ M	[110]

**Table 4:** Comparative study of the performances of the graphene-based sensors for the detection of ATM1 and AFB1 compounds.

Processed Materials	Fabrication Technique	Detection Analyte	Linear Range	Limit of Detection	Ref.
GO, AFB1 aptamer, TPE-Z	Stirring	AFB1	0–3 ng/mL	0.25 ng/mL	[112]
GO, ATM1 aptamer	Centrifugation	ATM1	0.2–10 µg/kg	0.05 µg/kg	[113]
mAb, Fe_3_O_4_, rGO	Ultrasonication, centrifugation	AFB1	-	50 ng/L and 3.8 ng/L	[114]
CdTe quantum dots, GO	Hummers’ method, stirring, annealing	AFB1	-	1 nM	[115]
Luminol, Ag NPs, GO, Fe_3_O_4_	Self-assembly, stirring	ATM1	5–150 ng/mL	0.01 ng/mL	[116]
rGO, ss-DNA	Hummers and Offman method	AFB1	R^2^ = 0.996	1 × 10^−10^–7 × 10^−8^ g/mL	[117]
α-cyclodextrin, GQDs, Ag NPs	Electrodeposition	ATM1	0.015 mM–25mM	2 μM	[120]
rGO nanosheets, Au nanorods, ITO	Self-assembly	AFB1	100 ng/mL–1 pg/mL	6.9 pg/mL	[122]

## Data Availability

Not applicable.
